# *Everybody Brush!* Consumer Satisfaction with a Tooth Decay Prevention Program

**DOI:** 10.3389/fpubh.2017.00264

**Published:** 2017-09-27

**Authors:** Joana Cunha-Cruz, Colleen E. Huebner, Sharity Ludwig, Jeanne Dysert, Melissa Mitchell, Gary Allen, R. Mike Shirtcliff, JoAnna M. Scott, Peter Milgrom

**Affiliations:** ^1^Department of Oral Health Sciences, University of Washington, Seattle, WA, United States; ^2^Department of Health Services, University of Washington, Seattle, WA, United States; ^3^Advantage Dental Services, LLC, Redmond, OR, United States; ^4^Research and Graduate Programs, University of Missouri—Kansas City, Kansas City, MO, United States

**Keywords:** child preschool, dental caries/epidemiology/*prevention & control, health promotion, toothpastes, fluorides

## Abstract

**Introduction:**

Twice-daily caregiver-supervised toothbrushing with fluoridated toothpaste is an effective and widely recommended strategy to prevent tooth decay in children. Qualitative research suggests that low-income caregivers know the recommendation but would benefit from toothbrushing supplies and advice about how to introduce this health behavior especially as the child becomes older and asserts autonomy to do it “myself.” Our objective is to assess consumer satisfaction with the evidence-based theory-informed campaign and usefulness of materials that were home delivered. The focus of the evaluation was families with children <36 months of age because of the high incidence of disease in this population.

**Methods:**

A dental care organization designed and implemented *Everybody Brush!* in three counties of Central Oregon. Participants were families of Medicaid-insured children <21 years of age. Participants were randomly assigned to one of the three study groups: test (supplies, voice/printed messages, telephone support), active (supplies), and a waitlist control. Program materials were in English and Spanish. Caregivers of children <36 months were interviewed at the beginning and end of the program.

**Results:**

A total of 83,148 toothbrushing kits were mailed to 21,743 families. In addition, 93,766 printed messages and 110,367 recorded messages were sent to half of the families. Caregivers were highly satisfied. On a global rating scale from 0 to 10 (worst to best program possible), they rated the program 9.5 on average (median: 10, SD 0.9). On a scale from 0 to 10 (not at all to very useful), mean ratings for usefulness of the toothbrushing supplies was 9.5 (SD = 1.5), for the printed postcard messages was 7.2 (SD 3.6), and for the voice telephone messages was 6.5 (SD 3.9).

**Discussion:**

A dental care organization carried out a complex community intervention designed to address excess tooth decay among low-income children. Caregivers were highly satisfied with the *Everybody Brush!* program and toothbrushing supplies were considered the most useful, followed by printed messages. Voice telephone messages were rated least useful. Further evaluation of the impact of the program on toothbrushing behavior and dental-care utilization is underway.

## Introduction

Widespread availability of fluoridated toothpaste is one reason the U.S. has seen a decline in tooth decay over the past several decades. Regular brushing with fluoridated toothpaste maintains a low level of fluoride in the saliva. The fluoride helps repair tooth surfaces demineralized by acid by-products from the dietary sugar metabolism of bacteria that colonize the teeth (1). Evidence of the benefits of twice-daily brushing of children’s teeth with fluoridated toothpaste (2) has led the American Academy of Pediatric Dentistry and other professional organizations to endorse this practice with children of all ages (3). Although the general public is aware of the recommendation to brush twice a day (4), not all vulnerable children actually benefit from this relatively simple health behavior. In a study of 1,021 African-American families in inner city Detroit, preschool children brushed their teeth about half as much as recommended (5). Maternal self-efficacy and knowledge were important predictors of brushing behavior (5). An international study of over 2,800 parents and their children also found that the single best predictor of a child being caries free at age 4 was parents’ perceived skill at regularly brushing their child’s teeth (1, 6).

Barriers to putting the toothbrushing recommendation into practice, especially for parents with limited access to pediatric dental care, include parents’ uncertainty about when to initiate brushing, the cost of toothbrushing supplies, and children’s resistance to brushing (4). The U.S. Food and Drug Administration’s rules regarding toothpaste labeling and consumer marketing has added to the confusion about what type of toothpaste to use for young children, and how much and when to begin using fluoridated toothpaste (2). Qualitative research with parents of low-income children identified their preference for the free distribution of toothbrushes and toothpastes, and advice of when to start brushing, amount of toothpaste and information on how to overcome barriers for regularly brushing a child’s teeth as ways to help them make a habit of brushing their children’s teeth twice a day.

Outside of the U.S., toothbrushing campaigns with free distribution of toothpaste and toothbrushes—at a child health visit and by mail—have showed positive effects on parents’ behaviors. Parents who participated in such a campaign, compared to those who did not, were significantly more likely to report twice-daily toothbrushing of their children (7). When accompanied by educational opportunities, these campaigns showed even more promising results to improve oral hygiene and decrease dental caries (8–11).

Mobile health (mHealth) is a logical approach to deliver health education and services. Because of its wide reach, small investment on time and money on the part of participants, mHealth is suitable to accompany broad-based public health campaigns. Most mHealth interventions for behavior change have focused on general health and well-being related behaviors such as parenting skills, physical activity, smoking cessation, sunscreen use, prevention of sexually transmitted diseases and diabetes (12), most of them on a small scale. Few mHealth studies have focused on oral health-related behaviors (11, 13–16). We identified two small-scale interventions specifically targeting toothbrushing behavior, one for young adults (13) and the other one for parents of young children (11).

While most mHealth studies delivered messages *via* text or video trough mobile phones (11–15), evaluation of the interventions with messages delivered *via* voice are scarce (12). Voice messages have the capacity to overcome low literacy barriers and to be easily relatable to the participants if the voice in the recorded messages are from local personalities. For example, the large majority of participants in a prevention program in Ghana elected to receive voice messages instead of text messages (17). Most mHealth reported improvements in the target behavior, but only three reported on consumer satisfaction and usefulness of supplies or mHealth components of the intervention (11, 18, 19).

Dental services for low-income children are publicly financed and the dental care organizations have the obligation to use these scarce resources wisely. Satisfaction with community intervention programs in oral health has not been reported. We designed and implemented a mHealth program called *Everybody Brush!* that included periodic delivery to families’ homes of fluoridated toothpaste, toothbrushes, and written advice along with printed and voice messages and a telephone helpline to answer caregivers’ questions and assist further with tips to carry-out twice a day brushing. The objective of the analyses reported here is to assess consumer satisfaction with the *Everybody Brush!* program and usefulness of the toothbrushing supplies and voice and print messages.

## Materials and Methods

### Design

The study design was a randomized controlled trial aimed to promote toothbrushing behavior and reduce tooth decay in children and adolescents from low-income families living in rural communities in Oregon. The protocol for the trial has been published (20). The protocol was submitted to the Institutional Review Board of the University of Washington for consideration. It was determined to not meet the definition of human subjects’ research.

### Program Implementation

#### Participants and Settings

The population was all families of children and adolescents (ages 1–21 years old) enrolled in Medicaid and assigned by the State of Oregon to a single dental care organization (Advantage Dental Services, LLC., Redmond, OR, USA) who resided in the three largely rural counties in Central Oregon (Crook, Deschutes, Jefferson). The counties, served by the Pacific Source Community Solutions Coordinated Care Organization, are part of a statewide Center for Medicaid and Medicare Transformation Project that provided the majority of the funds for the evaluation of the project. Medicaid enrollees represent 12–19% of all children ages 1 to <21 years old in these three counties. The median per capita annual income across the counties is US$50,000 to $60,000. Most homes are served by public water systems without fluoridation. The dental care organization has primary care dentists in individual rural practices and clinics throughout the three-county area.

#### Intervention Description

##### Theoretical Framework

The theoretical framework of *Everybody Brush!* reflected theories of behavior change and foci for behavioral change interventions, including those described by Fishbein et al. (21) and Michie et al. (22). We used the theoretical domains framework to map behavioral determinants of toothbrushing to behavior change techniques (23) that reflected our previous research that found parents of young children want practical advice to help establish toothbrushing routines (4). In keeping with parents’ preferences and self-described barriers, the intervention content operationalized the theoretical domains of external constraints, intentions, norms, and skills (15). For instance, we offered toothbrushing supplies to reduce environmental barriers for adopting and maintaining the behavior. We also offered information to reduce external barriers of time and place (e.g., messages such as “You can brush your child’s teeth while in the bathtub: it’s fun!”), knowledge of when to initiate the behavior (e.g., “Start brushing your child’s teeth with the very first tooth”), instruction for brushing a young child’s teeth (e.g., “Use a smear for kids younger than 2 years old”) and tips to overcome behavioral resistance. Messages for older children appealed to social norms (e.g., “Ugly teeth make an unhappy smile”) and were adjusted to the skills needed for children age 2 years and up as well as those ready for independent brushing (e.g., “Brush with a pea-size amount of toothpaste”). In keeping with behavioral change theory that recognizes there are individual differences in perceived barriers and enabling factors, and the restrictions inherent in a study of this size, we made no attempt to tailor the intervention messages to individual caregiver–child dyads. Rather, we used prior research findings and theoretical frameworks to identify the domains most likely relevant to caregivers of children engaged in this specific health behavior.

##### Intervention Materials and Content

Intervention materials consisted of supplies, voice, and printed messages and a telephone helpline with trained staff to promote twice a day toothbrushing with fluoride toothpaste. The print and voice messages and telephone scripts are described in the detail in the study protocol (20) and are available to others upon request.

###### Supplies

*Everybody Brush!* kits were delivered to the homes of participants every 3 months for 9 months. The kit contained toothbrushing supplies for the entire family (the average size was seven people) and one “cling sheet” that could be affixed to a mirror or other smooth surface. The toothbrushing supplies were 7 tubes of fluoridated toothpaste (0.85 oz.), 3 adult-size toothbrushes, 1 toothbrush each for a child ages 4–24 months, 2–4 years, and 5–7 years. The cling sheet contained instructions to brush twice a day with an age-appropriate amount of fluoride toothpaste depicted by illustration as well as information about how to request additional supplies. Instructional materials were provided in both English and Spanish and were written at 6th-grade reading level.

###### Voice and Printed Messages

Voice messages were delivered to participants *via* automated telephone calls and printed messages were delivered *via* mailed postcards every 2 weeks for 9 months, so that the voice and print messages were intercalated. The messages were written to reflect universal health literacy considerations (24), written at or below a 6th-grade reading level, excluded medical and dental jargon and included pictorial aids to assist with comprehension for instance, to show the correct amount of toothpaste to use with younger and older children (4, 25). The content prioritized increasing awareness of the value of toothbrushing and helping caregivers overcome challenges. The content of the messages reflected three sets of factors known to influence health behavior: strong intention, necessary skills, and the absence of environmental constraints that interfere with the behavioral intent (21).

###### Telephone Support

Bilingual (English and Spanish) culturally competent staff members trained in oral hygiene and behavioral change techniques were available to support families who used the help line to ask for additional support.

#### Assignment to Intervention Groups

For purposes of evaluation, participants were assigned to one of three study groups: a test, an active, and a waitlist control. The test group received the toothbrushing supplies, the voice and printed messages, and the telephone support. The active control group received only the toothbrushing supplies and the waitlist control group did not receive supplies or messages and were placed on a waiting list to receive the supplies after the end of the evaluation period.

Participants who met the inclusion criteria were randomly allocated to the groups using a computer-generated allocation schedule. First, we randomly allocated the 450 families with children less than 36 months who participated in the baseline interview to the three groups: test, active control, and waitlist control. We then randomly allocated with equal probability the remaining families to one of two groups: test or active control. The final sample size per group was 10,797 families in the test group, 10,796 in the active control group, and 150 in the waitlist control group.

### Program Evaluation: Consumer Survey

#### Selection and Recruitment of Participants

Caregivers of children less than 36 months of age were invited to participate in an interview before the implementation of the intervention. A list of the families meeting the inclusion criteria was randomly ordered and the caregivers were telephoned and invited to participate in an interview. Invitations were made until 450 caregivers agreed to participate. After the interview, the families were randomly assigned to one of the three groups. At the end of the intervention period, the same caregivers were re-contacted and invited to participate in an exit interview.

#### Data Collection and Instruments

Data were collected through telephone interviews conducted by bilingual staff (English, Spanish) from the customer service department of the dental care organization before the start of the intervention (baseline) and after the intervention period (exit interview). At least five attempts were made to reach the caregiver. Interviewers were unaware of caregivers’ group assignment.

Data on overall satisfaction with the *Everybody Brush!* program were collected using a single question in which the caregiver was asked to rate the program on a scale from 0 to 10, from worst to best program possible. Additionally, caregivers were asked if they and their children used the toothpaste and toothbrushes from the kit and if they received the print and voice messages. Usefulness of these sets of materials were assessed on a 11-point scale (0–10) from not at all useful to very useful in three questions: toothbrushing supplies, printed postcards messages, and voice telephone messages. The interview concluded with an open-ended question asking if there were anything to note about their feelings about the *Everybody Brush!* program.

#### Data Analysis

Descriptive statistics were used to describe implementation process, participants’ characteristics, and perceptions of the program. Continuous variables were expressed as means and categorical variables as frequencies and percentages. We compared caregivers’ ratings and reported use of the different program elements between the test and active control groups using Wilcoxon Rank Sum tests for continuous variables and Chi-square and Fisher exact tests for categorical variables at a statistical significance level of 0.05.

## Results

### Implementation Process

*Everybody Brush!* was implemented over a 9-month period from August 2014 through April 2015. The project manager from the dental care organization purchased the supplies from the toothbrush and toothpaste manufacturer and contracted with an outside fulfilment company to store, assemble, address, and mail toothbrush kits.

Over the course of the intervention, 83,148 toothbrushing kits were mailed to 21,743 families. The group of 10,797 families assigned to receive additional support (the test group) were sent a total of 110,367 pre-recorded voice messages *via* automated telephone calls and 93,766 printed postcards *via* mail. The toothbrushing kits were sent in August and November 2014 and February 2015. Postcards and telephone messages were delivered through April 2015. The helpline and other telephone service to request additional supplies were available throughout the intervention period from August 2014 through April 2015.

The overall direct cost of the intervention, exclusive of costs to design and evaluate the program, was US$ 42.50 per family in the test group and US$ 37.62 per family in the active control group over the 9 months. With about four Medicaid enrolled children per family, the cost per child ranged roughly from $9.41 to 10.60 per child per year. The overall cost includes the cost of the materials, fulfillment, freight, and postage of the toothbrush kits and graphic design and printing of the cling sheet. Additional costs for the test group include graphic design and printing of postcards, telephone messaging charges, and support for the advice line. For the test group, costs were US$ 5.41 per family for printing, addressing and mailing of postcards and US$ 1.48 per family for the telephone calls for the recorded voice messages. These costs do not include internal company resources required to establish and administer the program.

### Usefulness of Intervention Components

Of the subsample of 450 caregivers who interviewed at baseline, 223 (49.6%) completed the final interview. At the time of the final interview, 33% of children were 1 year old, 37% were 1 to 2 years old, and 30% were 3 years old. Caregivers interviewed were fairly typical of the Medicaid client population in Oregon: 26% of caregivers had less than a High School (HS) diploma, 26% had a HS diploma, and 48% had completed formal education beyond high school; 44% had three or more children less than 21 years old in the household.

Of the 153 participants in the test and active control groups, 90% reported that they received the kits. Of the participants in the test and active control groups who reported receiving the kits, approximately two-thirds of caregivers reported their children used the toothbrushes (68%) and the toothpaste (62%) from the kits. More than half said they or other family members used the adult toothbrushes (58%) or toothpaste contained in the kits (52%). Within the test group, 84% of the children were reported to have used the toothbrushes and 78% used the toothpaste, 70% of the caregivers said they used the toothbrushes and 61% used the toothpaste. Within the active control group, 95% of the children were reported to have used the toothbrushes and 89% used the toothpaste, 84% of the caregivers said they used the toothbrushes and 76% used the toothpaste. Differences between groups were not statistically significant.

Table 1 gives the ratings of usefulness of program components. When asked to rate the usefulness of the toothbrushing supplies overall, on a scale from 0 (not at all useful) to 10 (very useful), caregivers gave a rating of 9.5 on average (SD = 1.5). The usefulness of the supplies was rated 9.6 (SD = 1.2) by the test group and 9.4 (SD = 1.8) by the active control group. Because the data were highly skewed, we examined their ratings as a dichotomous variable and compared the test and active control groups. Eighty-one percent of the test group and 91% of the active control group rated the supplies as 7 or above. Differences in means and proportions between groups were not statistically significant.

**Table 1:** Usefulness of the intervention materials and satisfaction with the *Everybody Brush!* program.

	Test group*			Active control group*			Total		
			
	N	Mean	SD	*N*	Mean	SD	*N*	Mean	SD
**Usefulness of**									
Toothbrushing supplies	61	9.6	1.2	76	9.4	1.8	137	9.5	1.5
Printed postcard messages	54	7.2	3.6						
Voice telephone messages	49	6.4	3.9						
Overall satisfaction	61	9.5	1.1	76	9.5	0.9	137	9.5	0.9

*Wilcoxon Rank Sum tests for comparison between groups: p > 0.05.

Caregivers in the test group (the group that received supply kits and additional support) were asked to rate the usefulness of the postcards and telephone messages. On a scale from 0 (not at all useful) to 10 (very useful), caregivers rated the postcards on average a 7.2 (SD = 3.6). The telephone messages were rated lower, 6.4 (SD = 3.9), on average. Sixty-seven percent rated the usefulness of the postcards as a 7 or above, and 59% rated the usefulness of the telephone messages as a 7 or above.

### Consumer Satisfaction with the *Everybody Brush!* Program

Using a global rating scale from 0 (worst) to 10 (best program possible), the average rating by caregivers was 9.5 points (median: 10, SD: 0.9). The satisfaction with the program was rated 9.5 (SD = 1.1) by the test group and 9.5 (SD = 0.9) by the active control group. We found 97% of the test group and 99% of the active control group gave a favorable rating of 7 points or higher. The average rating by caregivers in the test and active control groups were not statistically significant.

Sixty-six caregivers offered a response to the open-ended question asking their opinion of *Everybody Brush!* program. Nine caregivers suggested items to add to the supply kit; one said the supply kit contained too many items. Three thought that the telephone calls or postcards were not useful. The majority (50 of 66) shared very positive comments. Most comments were of general praise (i.e., “good program”) and others reflected favorably on the dental care organization specifically. Sample comments are provided in Table 2.

**Table 2:** Opinion of participants about the *Everybody Brush!* program.

“I love to get these supplies because it gets expensive buying these all the time.” *“It’s good you* (dental care organization) *worry about children’s teeth.”* “It’s a good help you offer and the information as well.” “I just want to thank you for this program.” “It’s been really good. It helps parents brush their child’s teeth.”

## Discussion

*Everybody Brush!* was a family-level mHealth effort with free distribution of fluoridated toothpaste and toothbrushes to address gaps in knowledge and self-efficacy about toothbrushing among families with low-income children. Consumers (parents) were very satisfied with the program and considered the supplies useful. Consumers rated the toothbrushing supplies more highly than the written oral health promotion messages, and the telephone messages were rated lowest of all.

Studies reporting on consumer satisfaction from mHealth health promotion campaigns are scarce. Previous studies on the perceptions of consumers with dental services have been focused on dental care received at dental practices (26–31). Few studies have reported caregivers’ experience with preventive care for their children, and even these only asked about care received at medical offices (32) or at dental practices (29–31).

The studies on health promotion campaigns with free distribution of toothbrushing supplies reported positive results on oral health outcomes (7–10) and behavioral changes (9, 11), but only one small scale program reported on consumer satisfaction (11). This program delivered a kit that contained a toothbrush, a book, a brushing chart with stickers, and a mirror cling at the beginning of the program and twice weekly text messages with a link to a video for 5 weeks. Satisfactions with the materials were 4.5 on a 1–5 scale and 88% of the caregivers indicated they were satisfied or very satisfied with the multimedia text messages. These results are similar to the ratings of the toothbrushing supplies and overall satisfaction from our program. Understanding participant experiences within large-scale oral health promotion programs may help improve the reach and impact of these programs.

Implementation of this large-scale health promotion program was challenging not only to the dental care organization. Assembly of the kits was outsourced to a fulfillment center after the initial kit was completed within the dental service organization itself and proved to require resources beyond the capability of the organization. In response, the organization had to identify and hire a fulfillment center to complete the assembly of the remaining kits. The postal system in the three rural counties was slowed by the large number of kits and postcards that had to be delivered to homes in this intensive campaign. There were also challenges in carrying out the evaluation at the end of the program. After the implementation period, 12 months from its initiation, many household’s phone numbers were no longer in service. Other parents with working numbers were not successfully interviewed despite the repeated attempts.

*Everybody Brush!* was well received by the dental care organization and its members and the value of the lessons learned were perceived to outweigh the implementation challenges. As a quality improvement project, the organization had the opportunity to collaborate with university researchers, health departments, and community organizations. It also used in-house resources including a project coordinator, the communication and graphic arts staff, and the customer service staff who were trained to provide social support and advice to families asking for additional tips to overcome barriers to toothbrushing. Customer service staff members were responsible for conducting the telephone interviews used for evaluation. The use of bilingual and multicultural staff and materials and the delivery of supplies strengthened the commitment to population health and reduction of rural and ethnic disparities in health by serving families at their homes and using communication in both English and Spanish.

Community-based primary prevention has largely been seen as separate and apart from the U.S. dental-care delivery system. With the expansion of Medicaid and evolution of capitation payment by states, however, dental care organizations are being required to care for a panel of clients and assume full risk for the cost of defined care benefits. These organizations can use global budgeting to allocate resources for primary prevention programs like the *Everybody Brush!* program. The cost per child was about $10. To put this in perspective, 52% of the children 6–9 years of age have had at least one cavity and 20% have untreated decay in this population (33). The cost of providing a single filling or tooth extraction can easily cost 4–10 times as much as the per capita cost of the program. The additional costs incurred for the printed and voice messages were not high, but participants seemed to prefer the printed postcards rather than the voice messages. Of course, toothbrushing with fluoridated toothpaste does not prevent all cavities and the contact with the community program may drive new demand for treatment and increased initial treatment costs among those who previously were non-users. Nevertheless, overall money costs may be less because more complex treatment and pain and suffering are averted.

In this quality improvement program, the dental care organization adapted an environmental and mHealth strategy shown to increase the adoption of the behavior and improve health outcomes (7–10). Further evaluation of the *Everybody Brush!* program will examine toothbrushing behavior, utilization of care, and oral health associated with the program. In conclusion, consumers were satisfied with the program, particularly with the free distribution of the toothbrushing supplies.

## Ethics Statement

Consistent with U.S. Federal regulations, this quality improvement project did not meet the definition of research and therefore program participants are not considered research subjects. University of Washington personnel have access only to evaluation data that contain no information that could identify individual participants.

## Author Contributions

The quality improvement project was conceived and designed by J-CC, CH, SL, JS, JD, MM, GA, RS, JS, and PM and all of the authors contributed to the data collection, analysis, and interpretation; contributed to the synthesis of the findings reported here, critically revised the manuscript for important intellectual content, approved the final version, and agreed to be accountable for all aspects of the work in ensuring that questions related to the accuracy or integrity of any part of the work are appropriately investigated and resolved.

## Conflict of Interest Statement

SL, JD, MM, GA, and RS are employed by Advantage Dental Services, LLC. All other authors declare no competing interests. This quality improvement project was funded by Pacific Source Community Solutions, Inc., and Advantage Dental Services, LLC. Pacific Source Community Solutions, Inc. was not involved in the quality improvement project design or collection, analysis, or interpretation of the evaluation data.
